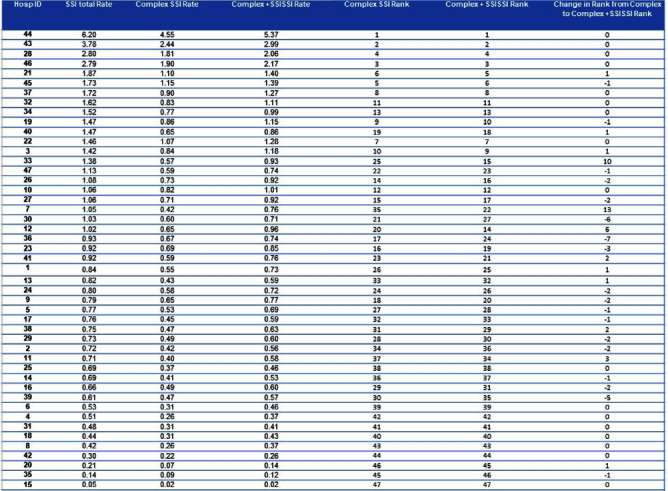# A Deeper Look at Proposed Surveillance of Superficial Incision Surgical Site Infections (SSISSIs)

**DOI:** 10.1017/ash.2024.315

**Published:** 2024-09-16

**Authors:** Jessica Seidelman, Brittain Wood, Polly Padgette, Valerie Payne, Linda Crane, Linda Roach, Deverick Anderson

**Affiliations:** Duke University; Duke; Duke Infection Control Outreach Network; Duke University Medical Center; Duke Ic Outreach Network; Duke Center for Antimicrobial Stewardship and Infection Prevention

## Abstract

Although the National Healthcare Safety Network (NHSN) recommends the reporting of superficial incisional SSIs, the standardized infection ratio (SIR) models used by the NSHN exclude superficial incisional SSIs cases. Yet, some superficial incisional SSIs may lead to serious adverse patient outcomes. We previously proposed a new category of such infections: serious superficial incisional surgical site infections (SSISSIs), defined as a superficial incisional SSI that (1) required debridement in an operating room and/or (2) led to a hospital readmission within 30 days of surgery. The objective of our study was to determine the prevalence of SSISSIs in a large network of community hospitals and compare hospital rankings of SSI rates of organ/space and deep SSIs (complex SSIs) with and without SSISSIs. We performed a retrospective descriptive analysis of prospectively collected data on 35 NHSN surgical categories in 47 community hospitals within the Duke Infection Control Outreach Network (DICON) from 1/1/2013-12/31/2022. All hospitals used standardized surveillance and data collection strategies throughout the study period. The Wilcoxon rank-sum was used to test for differences in performance rankings of hospitals sorted by rates of complex SSIs alone compared to complex and SSISSI rates. A two-tailed P value of .05 or less was considered significant. Overall, 11,617 SSIs occurred after 1,272,257 surgeries (0.91 SSIs/100 procedures). Out of 3,996 superficial SSIs, 2,038 (17.5% overall, 51.0% of superficial incisional) met criteria for SSISSI. 112 (5.5%) were diagnosed during the current admission and required takeback to the OR for infection; 1,926 (94.5%) were diagnosed during a readmission; and 3841 (33.1%) were diagnosed during readmission and returned to the OR. (Table1) The highest proportion of SSISSIs was diagnosed in patients who underwent gastrointestinal surgery (32.0%) or orthopedic surgery (24.0%). (Table2) Performance ranking of individual hospitals based on rates of complex SSIs, differed significantly when including SSISSIs (p= 0.02). (Table3) Discussion Our findings suggest that SSISSIs make up a moderate but important proportion of SSIs in community hospitals. SSISSIs can be identified through established database surveillance looking at objective measures of returning to the OR for debridement and/or readmission within 30 days. Hospital rankings differed significantly when SSISSIs were added to complex SSIs to calculate SSI rates. As such, including SSISSIs likely provides a more accurate depiction of SSIs with important outcomes and is not as subjective to surveillance bias. Next steps would be specifically to look at outcomes data for complex SSIs compared to SSISSIs to fully evaluate the

**Disclosure:** Jessica Seidelman: UptoDate- Section Editor; BoneSupport- Consultant

**Figure:**
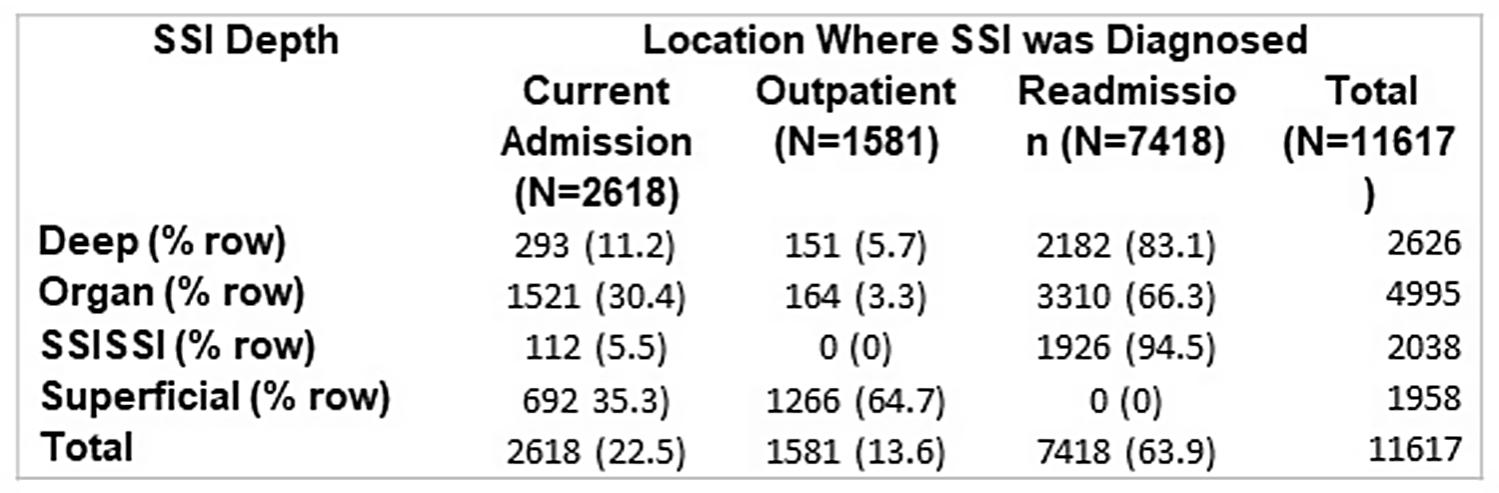

**Figure:**
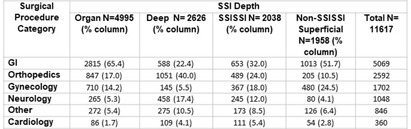

**Figure:**